# Monitoring of Antarctica’s Fragile Vegetation Using Drone-Based Remote Sensing, Multispectral Imagery and AI

**DOI:** 10.3390/s24041063

**Published:** 2024-02-06

**Authors:** Damini Raniga, Narmilan Amarasingam, Juan Sandino, Ashray Doshi, Johan Barthelemy, Krystal Randall, Sharon A. Robinson, Felipe Gonzalez, Barbara Bollard

**Affiliations:** 1 School of Electrical Engineering and Robotics, Faculty of Engineering, Queensland University of Technology, Brisbane City, QLD 4000, Australia; damini@majans.com (D.R.); narmilan.amarasingam@hdr.qut.edu.au (N.A.); felipe.gonzalez@qut.edu.au (F.G.); 2 Securing Antarctica’s Environmental Future (SAEF), Queensland University of Technology, Brisbane City, QLD 4000, Australia; 3 Securing Antarctica’s Environmental Future (SAEF), University of Wollongong, Wollongong, NSW 2522, Australia; ashraydoshi@gmail.com (A.D.); jbarthelemy@nvidia.com (J.B.); krandall@uow.edu.au (K.R.); sharonr@uow.edu.au (S.A.R.); bbollard@uow.edu.au (B.B.); 4 School of Earth, Atmospheric and Life Sciences, University of Wollongong, Wollongong, NSW 2522, Australia; 5 NVIDIA, Santa Clara, CA 95051, USA

**Keywords:** antarctic specially protected area (ASPA), machine learning, gradient boosting, convolutional neural network, unmanned aerial vehicle (UAV), lichen, moss, antarctic

## Abstract

Vegetation in East Antarctica, such as moss and lichen, vulnerable to the effects of climate change and ozone depletion, requires robust non-invasive methods to monitor its health condition. Despite the increasing use of unmanned aerial vehicles (UAVs) to acquire high-resolution data for vegetation analysis in Antarctic regions through artificial intelligence (AI) techniques, the use of multispectral imagery and deep learning (DL) is quite limited. This study addresses this gap with two pivotal contributions: (1) it underscores the potential of deep learning (DL) in a field with notably limited implementations for these datasets; and (2) it introduces an innovative workflow that compares the performance between two supervised machine learning (ML) classifiers: Extreme Gradient Boosting (XGBoost) and U-Net. The proposed workflow is validated by detecting and mapping moss and lichen using data collected in the highly biodiverse Antarctic Specially Protected Area (ASPA) 135, situated near Casey Station, between January and February 2023. The implemented ML models were trained against five classes: Healthy Moss, Stressed Moss, Moribund Moss, Lichen, and Non-vegetated. In the development of the U-Net model, two methods were applied: Method (1) which utilised the original labelled data as those used for XGBoost; and Method (2) which incorporated XGBoost predictions as additional input to that version of U-Net. Results indicate that XGBoost demonstrated robust performance, exceeding 85% in key metrics such as precision, recall, and F1-score. The workflow suggested enhanced accuracy in the classification outputs for U-Net, as Method 2 demonstrated a substantial increase in precision, recall and F1-score compared to Method 1, with notable improvements such as precision for Healthy Moss (Method 2: 94% vs. Method 1: 74%) and recall for Stressed Moss (Method 2: 86% vs. Method 1: 69%). These findings contribute to advancing non-invasive monitoring techniques for the delicate Antarctic ecosystems, showcasing the potential of UAVs, high-resolution multispectral imagery, and ML models in remote sensing applications.

## 1. Introduction

Due to the extreme climate conditions, Antarctica’s terrestrial ecosystems are commonly dominated by moss and lichen vegetation. Vegetation is restricted to ice-free areas and is consequently distributed primarily in coastal regions and inland nunataks where ice-free land is available [1]. The Windmill Islands coastline in East Antarctica is home to some of the largest “moss forests” on the continent. These moss forests experience extremes of temperature, light and water [2,3,4]. The growth and health of Antarctic moss beds relies heavily on the availability of liquid melt water from snow and ice, which is unreliable from year to year and over the course of the summer season [3]. The supply of liquid water is likely to become more unreliable for Antarctic mosses under continuing climate change, as snow banks retreat and precipitation patterns change [5]. Long-term monitoring has revealed a regional drying trend in the Windmill Islands that has resulted in changes in moss community assemblages and moss health in this region [6]. Specifically, as the region dries, green healthy moss becomes red and brown and finally turns black and becomes encrusted with lichens [4]. Remote monitoring techniques are required in order to accurately map and monitor changes in these ecosystems [4].

Traditional remote sensing, particularly satellite-based, has been instrumental over the decades but is now complemented by the emergence of consumer grade advanced sensors mounted on Unmanned Aerial Vehicles (UAVs) offering unprecedented detail [7,8,9]. This transition marks a significant advancement in monitoring techniques, with UAVs providing centimetre scale spatial resolution [10]. UAVs have been used in remote sensing to detect and segment numerous types of objects and different environments, such as agricultural fields, urban areas, forests, and bodies of water, providing valuable data for various applications including environmental monitoring, disaster management, and infrastructure inspection [11,12,13,14,15,16,17]. These technological tools, often accompanied by classical machine learning (ML) and deep learning (DL) methodologies, enhance the accuracy and efficiency of different vegetation mapping [18,19,20,21,22,23]. Studies have demonstrated the effectiveness of employing DL for accurately monitoring and classifying these delicate species like moss and lichen in diverse environmental settings [24,25,26].

The spatial-temporal monitoring of the Antarctic ecosystem has seen a shift towards remote sensing as an alternative to conventional methods, particularly with the widespread availability of high-resolution satellite imagery [10,27,28,29,30]. In particular, the last decade has witnessed an increase in UAV use in Antarctic research, offering a unique perspective on monitoring and mapping of vegetation [29,31,32,33]. In recent years, the integration of UAVs equipped with RGB, multispectral, and hyperspectral cameras have revolutionised vegetation mapping in Antarctica [34,35,36,37,38]. Mapping vegetation in the Antarctic environment through remote sensing techniques is constrained by the diverse and uneven nature of its surface coverage. This variability includes sparse vegetation, isolated individuals scattered amidst soil and rocks, small communities forming biocrusts on soil or rock, and more extensive vegetation mats in larger communities [10]. Such variability makes drones an attractive option due to the high resolution of imagery compared to satellites, but also means fewer studies have integrated UAV and AI for monitoring and mapping vegetation in the Antarctic environment [27,28,39]. In addition, only a limited number of studies have incorporated multispectral sensors to enhance the precision and accuracy of vegetation mapping in various environments [40,41].

Limited attention has been paid to the application of UAV multispectral data in classifying moss health classes including healthy moss, stressed moss, and moribund moss using DL techniques. Gaps persist, including the limited use of multispectral imagery and especially the need for sufficient ground truth data for robust modelling. This research addresses these gaps by employing high-resolution RGB and multispectral UAV data and leveraging AI for automatic classification and mapping of moss health in Antarctica. The primary objectives of this project were to develop and validate a methodology applicable to fragile environments, with a specific focus on identifying health risks and supporting conservation initiatives. The project aimed to assess the effectiveness of two ML methods for semantic segmentation of drone remote sensing data: (1) Extreme Gradient Boosting (XGBoost), a decision tree-based classifier; and (2) U-Net, a convolutional neural network (CNN)-based classifier. This paper provides additional validation for the workflow presented, as it now encompasses the processing of multispectral data obtained during the data collection campaign [39]. In summary, this research contributes to the evolving landscape of Antarctic vegetation monitoring by utilising advanced UAV technologies and AI methodologies. By addressing existing gaps, particularly in multispectral data usage and ground truth validation, the project aims to enhance the accuracy and effectiveness of monitoring changes in Antarctic moss health in the face of climate change.

The methods are presented in two sections. Section 2 focuses on data collection and curation, while Section 3 investigates ML models designed for vegetation mapping. Results are presented in Section 4, followed by discussion (Section 5), and conclusion and recommendations for future work in Section 6.

## 2. Data Collection and Curation

**Figure 1:**
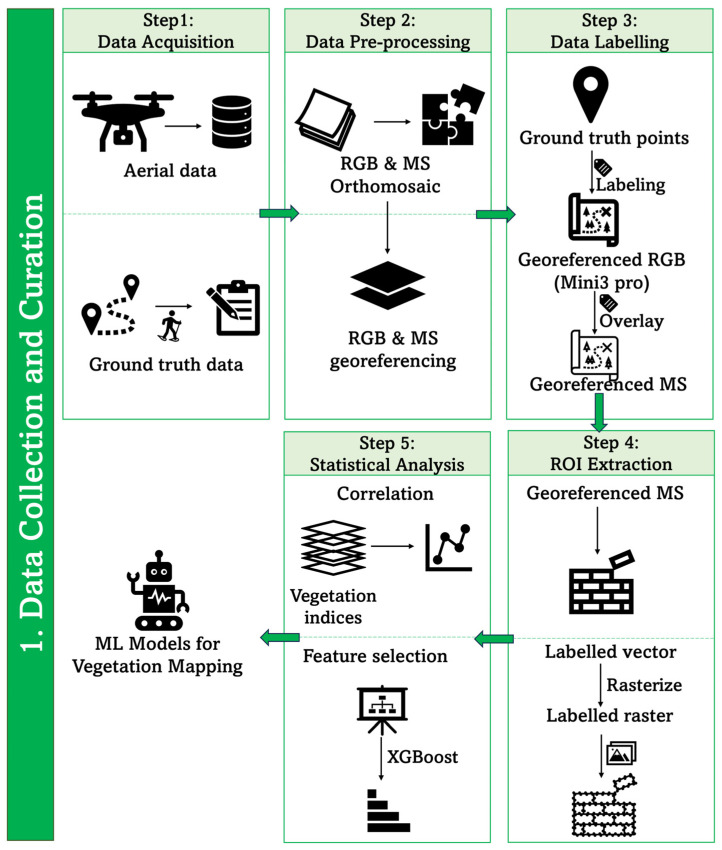
Processing pipeline of data preparation for classification of health status of moss and lichen in Antarctic environment.

Figure 1 depicts the proposed process pipeline used to prepare the data for developing the segmented maps using both XGBoost and U-Net classifiers. This process is broken down into five components: data acquisition, data pre-processing, data labelling, region of interest (ROI) extraction, and statistical analysis.

- Step 1: Data Collection

### 2.1. Study Area

**Figure 2:**
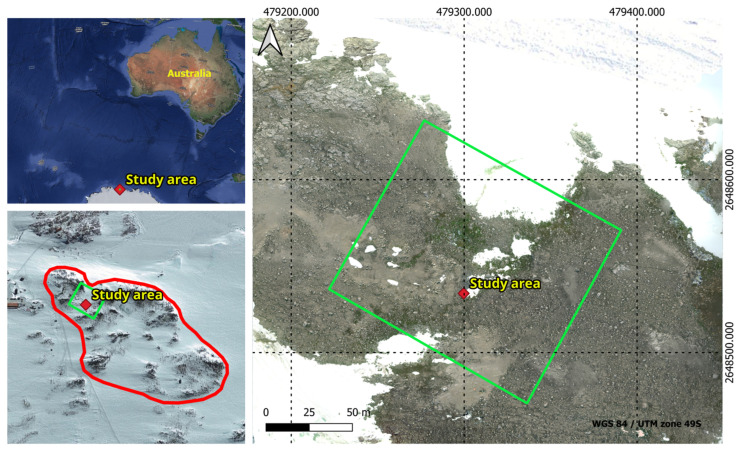
Geographical representation of ASPA 135 outlined by the red polygon, and the study area delineated by the green polygon on the map.

This study was conducted within Antarctic Specially Protected Area (ASPA) 135 (66°16′60″ S, 110°32′60″ E), located in the Windmill Islands region of East Antarctica as seen in Figure 2. This area is extremely rich in moss and lichen communities and spans an estimated area of 0.28 km^2^. The ASPA was visited three times between 2 January and 2 February 2023. Trips to the ASPA were restricted to periods between 3:30 and 6:30 pm (UTC+9) when light conditions were optimal. The average temperature during these visits was −2 °C, with typical wind gusts measuring 5.14 m/s.

### 2.2. Airborne Data Acquisition

Aerial data collection was carried out using a custom built BMR3.9RTK UAV developed by SaiDynamics Australia (Gold Coast, Australia). This quadrotor UAV, designed for extreme conditions, accommodates a multi-sensor payload of up to 7 kg (Table 1). The multi-sensor payload comprises a MicaSense Altum (AgEagle, Wichita, KS, USA) multispectral camera and a Sony Alpha 5100 (Sony Group Corporation, Tokyo, Japan) high-resolution RGB camera (Table 2). The ASPA was surveyed with the BMR3.9RTK using lawnmower patterns with an above ground level (AGL) height of 70 m. Imagery side and front overlap used was 80%, and the horizontal speed was 3.6 ms^−1^. Ground sampling distances (GSD) of multispectral and RGB sensors were 3.2 and 1.5 cm/pixel, respectively. In addition to this, the DJI Mini 3 Pro was used to get ultra-high resolution RGB data (Table 1).

### 2.3. Ground Truth Data Collection

The ground truth data collection involved the collection of accurate geolocation data of moss within the study site exhibiting various health states, from healthy to stressed and moribund. Health states were classified through colour changes of moss [42]. Table 3 details the criteria defining moss health and outlines the process by which judgments were made based on colour observations.

**Figure 3:**
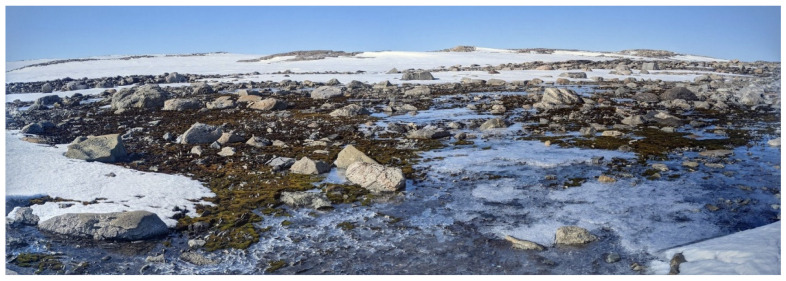
Ground image of study area depicting distribution of moss and lichen vegetation.

**Figure 4:**
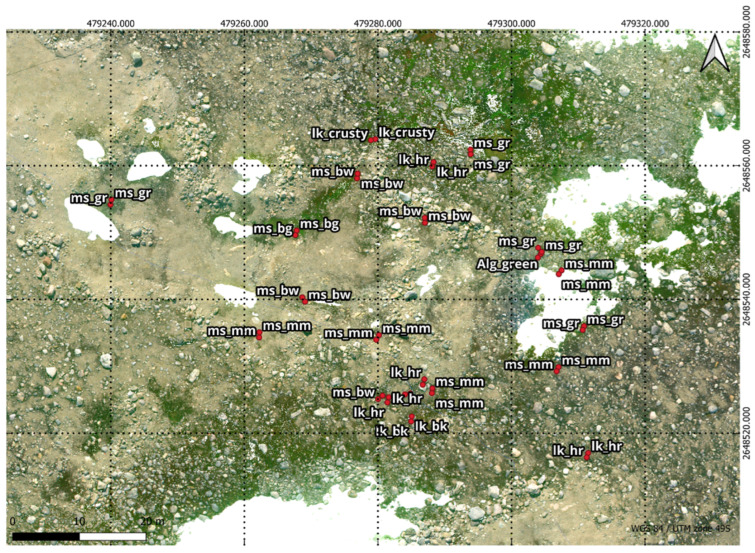
Ground truth points overlaid on RGB Orthomosaic: Healthy moss, characterised by a green colour, is labelled as ms_gr & ms_bg. Stressed moss, displaying shades of orange or red, is denoted as ms_rd. Moribund moss, with brown or black hues, is identified by the labels ms_bw & ms_mm. Featured lichen, include those with “hairy” (fructicose *Usnea* spp.) black, and crusty (crustose) attributes, were classified using the labels lk_hr & lk_bk & lk_crusty.

In conjunction with the UAV footage captured, the team collected 68 ground truth points on foot. GNSS RTK data was taken at ground truth points to georeference the UAV data. An industry standard Trimble GNSS system was used ensuring up to 2 cm accuracy on all ground truth points. By combining this high-precision GNSS technology with ground control points and the UAV’s RTK feature, the approach guaranteed precise alignment between aerial imagery and ground scans. This comprehensive strategy not only adhered to industry standards but also demonstrated a commitment to achieving the highest level of accuracy in geospatial data acquisition for the study. Figure 3 shows the study area and in Figure 4, the accurate depiction of ground truth points is highlighted as they overlay seamlessly on the RGB Orthomosaic, providing a visual reference for precise labelling.

- Step 2: Image Pre-Processing

**Figure 5:**
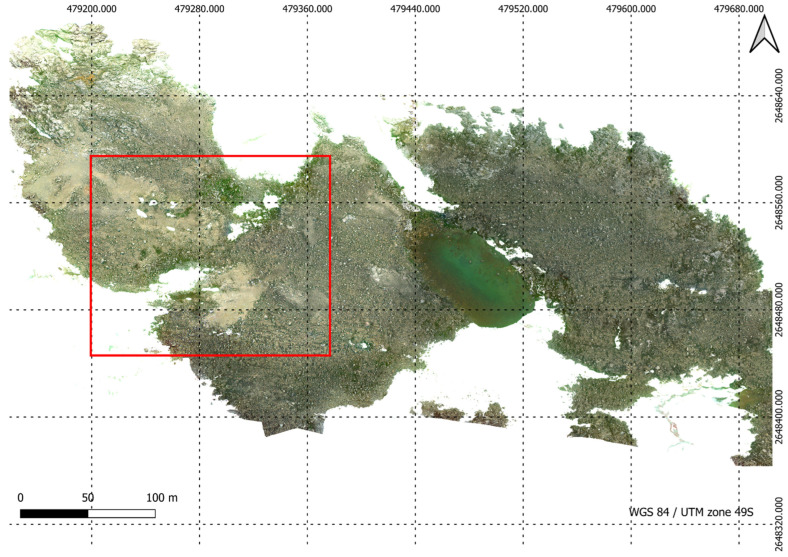
High resolution RGB orthomosaic of ASPA 135 developed from Sony Alpha 5100 raw images.

**Figure 6:**
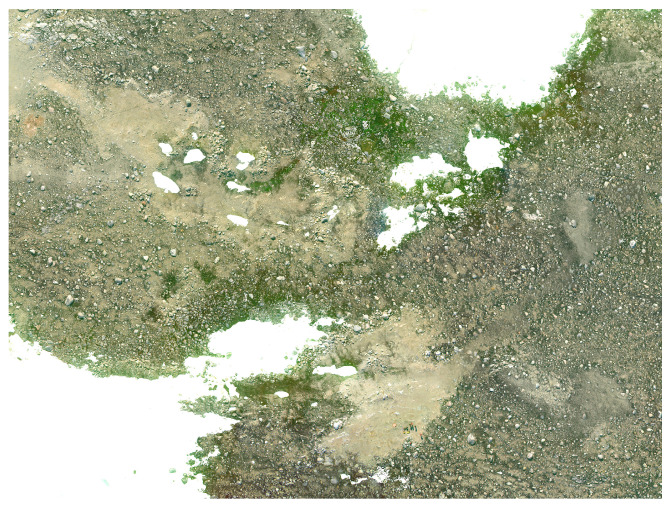
A region of high resolution RGB orthomosaic.

**Figure 7:**
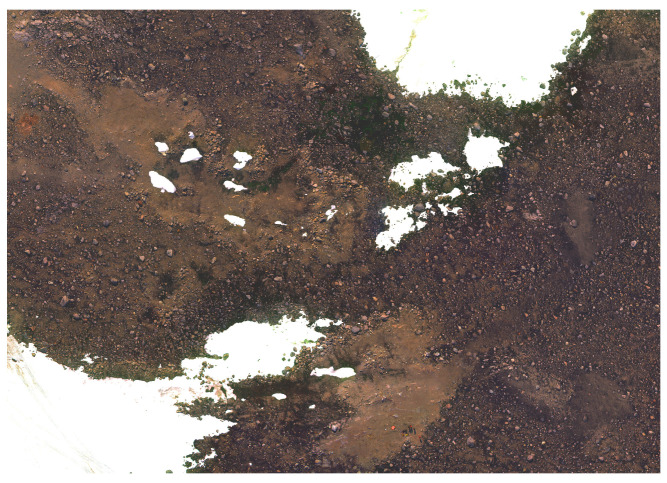
A region of high resolution multispectral orthomosaic.

The raw footage from the cameras was transformed into geometrically rectified images called orthomosaics. Image orthomosaics were obtained using Agisoft Metashape 1.6.6 (Agisoft LLC, Petersburg, Russia). The multispectral orthomosaic error metrics include X error (0.800178 metres (m)), representing the deviation in the longitudinal dimension (east-west direction); Y error (0.501047 m), indicating the error in the latitudinal dimension (north-south direction); and Z error (1.07776 m), representing the error in the altitude dimension. Additionally, the XY error (0.944104 m) signifies the combined error in both longitudinal and latitudinal directions, while the total error is reported as 1.4328 m. These values collectively provide a comprehensive understanding of the spatial accuracy of the orthomosaic, reflecting the accuracy of the georeferencing process for the multispectral imagery. After completing the orthomosaic generation, georeferencing was performed using the image registration tool in ArcGIS Pro 3.1.2 (Esri, Redlands, CA, USA). A 2nd order polynomial transformation method was applied, employing eight ground control points to enhance precision. Indicators of the georeferencing quality were provided through root mean square (RMS) errors. The forward transformation yielded a value of 0.005452, the inverse transformation recorded 0.005476, and the combined forward-inverse transformation exhibited 0.000003. The observation of lower RMS errors implies a heightened accuracy in aligning the imagery with the Earth’s surface. This critical information ensures that the spatial data extracted from the datasets maintains accurate geographic positioning. Figure 5 showcases a high-resolution RGB image created using the Sony high-resolution RGB Camera. Figure 6 and Figure 7 show a region (red colour polygon in Figure 5) of high resolution RGB imagery and georeferenced multispectral region of interest, respectively.

- Step 3: Data labelling

**Figure 8:**
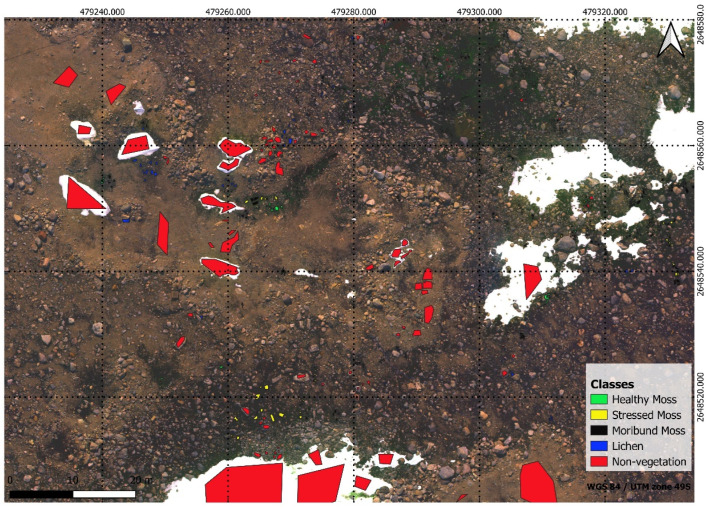
Labelled polygons with different classes over georeferenced multispectral orthomosaic.

The RGB and multispectral orthomosaics were utilised for labelling (Figure 8). The ground truth data was overlayed on the orthomosaics using QGIS (Version 3.2.0; Open-Source, Geospatial Foundation, Chicago, IL, USA) which is free and open-source geographic information software. Using the ground truth data, five classes were assigned for the segmented maps. In ASPA 135, labelling was applied to moss with varying health levels (Table 3) as well as three lichen types (Figure 4 legend). The class list for segmented vegetation consists of Healthy Moss, Stressed Moss, Moribund Moss, Lichen, and Non-Vegetation, assigned IDs from 1 to 5 respectively [4]. Polygons were manually drawn around the pixels within the ground truth quadrant that were associated to the ground truth label by checking the high resolution RGB imagery. After labelling, the labelled vector data was converted to a raster format, resulting in the generation of a labelled mask, through the utilisation of the rasterising tool in QGIS.

- Step 4: ROI extraction

The whole multispectral orthomosaic (13,659 × 6453 pixels) was cropped into a smaller region of interest (ROI) with 6004 × 4499 pixels based on the ground truth locations to train the XGBoost model, and it was further tiled into 15 smaller different dimensions of tiles (Dimension between 400 × 400 pixels and 900 × 700 pixels) to train the U-Net model and reduce computational complexity. This approach enabled the U-Net model to concentrate on localised data for alignment, enhancing efficiency and accuracy in the process. Additionally, labelled masks corresponding to the ROIs were extracted, each sharing the same dimensions as the multispectral ROI, to facilitate model training.

- Step 5: Statistical analysis

Correlation analysis was performed between moss health and twenty-one spectral indices as listed in Table 4 and four statistical features including mean, variance, skewness, and kurtosis. Following the correlation analysis, the estimation of feature importance was conducted using XGBoost, using spectral indices, all bands from multispectral imagery (blue, green, red, red edge, NIR, and thermal), and all the statistical features mentioned above.

## 3. Machine Learning Models for Vegetation Mapping

**Figure 9:**
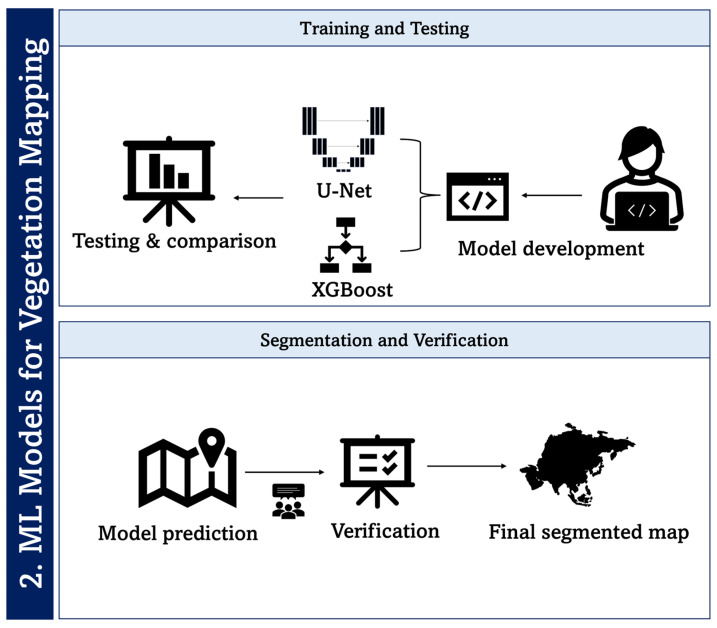
Processing pipeline of machine learning for classification of health status of moss and lichen in an Antarctic environment.

Once the training data was prepared, it was then fed into the ML classifiers. The training phase of the model was carried out using Python 3.8.10. For data processing and ML tasks various libraries were utilised, including Geospatial Data Abstraction Library (GDAL) 3.0.2, XGBoost 1.5.0, Scikit-learn 0.24.2, OpenCV 4.6.0.66, and Matplotlib 3.8.2. The training for the U-Net model was performed in Google Colab, which is equipped with a graphics processing unit (NVIDIA T4 GPU, NVIDIA: Santa Clara CA, USA). Figure 9 depicts the proposed process pipeline for ML model training for developing segmented maps using both XGBoost and U-Net classifiers.

### 3.1. XGBoost Model Training and Fine Tuning

**Figure 10:**
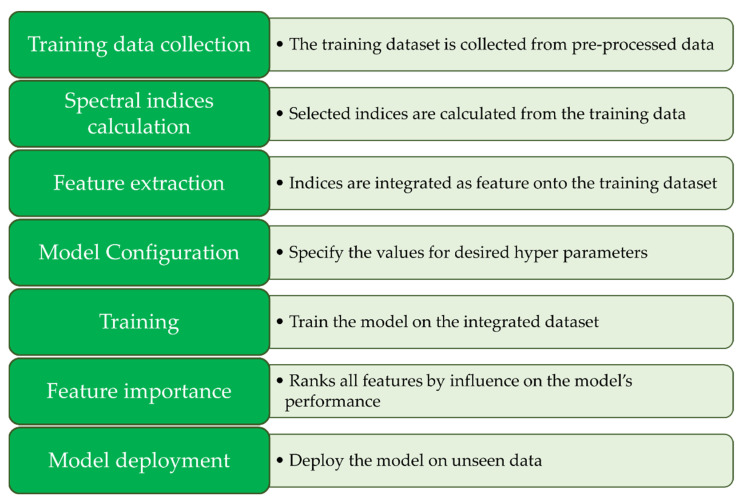
Processing pipeline employed for XGBoost segmentation, showcasing the sequential steps and pivotal components involved in the methodology.

XGBoost stands for extreme gradient boosting library [60], and it is designed to be a highly efficient, flexible, and portable ML algorithm. It is a state-of-the-art classifier that implements parallel tree boosting. It is known for its high execution speed and good performance. XGBoost was chosen for this project as it is a popular ML framework that is used widely in industry and academia, especially for detecting different vegetation types [61,62]. The XGBoost classifier script was fed the ROI multispectral and mask files described in the XGBoost ROI extraction section. The XGBoost model followed the pipeline described in Figure 10.

First, the ROI was loaded in and then the desired spectral indices were calculated. These spectral indices help the classifier to see data without the distortion of shadows or other anomalies. These indices become features in the training data set. Hyperparameter tuning for the XGBoost model was regarded as a critical aspect of the analysis. The optimal configuration, encompassing a maximum depth of 10, a learning rate of 0.02, 250 estimators, a subsample rate of 0.8, and a colsample_bytree of 0.8, was determined. Additionally, hyperparameters such as gamma, reg_alpha, and reg_lambda were systematically set to 0.0, 0.0, and 1.0, respectively. The entire process was carried out to ensure the model’s effectiveness and robustness, and the outcomes of this methodological approach are detailed in the subsequent sections. After completion, XGBoost conducts feature importance analysis to identify the features that exert the most influence on the model’s predictions. Subsequently, the trained model is validated using the test data.

### 3.2. U-Net Model Training and Fine-Tuning

**Figure 11:**
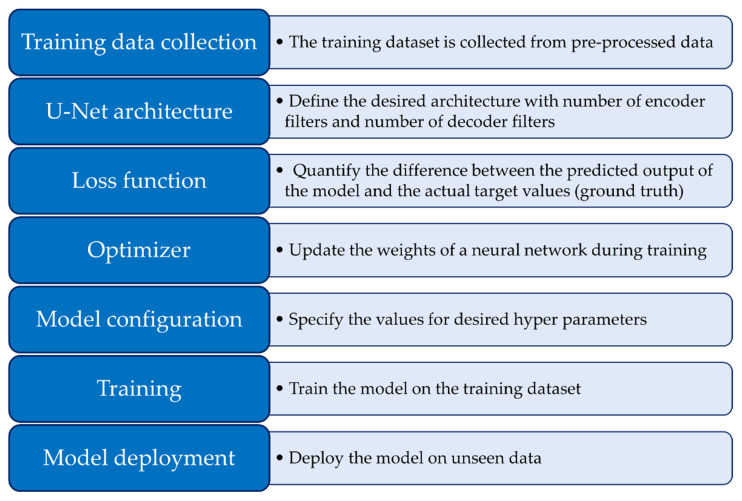
Processing pipeline employed for U-Net segmentation, delineating the sequential steps and critical components integral to the methodology.

U-Net is a network architecture known for its U-shaped encoder-decoder structure [63]. The architecture is simple, efficient, and widely used for semantic segmentation tasks. The 15 tiles, specified in the step 4 were uploaded to Google Drive so they could be accessed by the Google Colab script. Alongside the tiles, the labelled polygon file was rasterised and cropped to be the same size as each ROI. A custom U-Net architecture was designed for this project. Figure 11 demonstrates the pipeline of the U-Net model.

The process involves cropping the collected data into tiles of a predetermined size, employing the U-shaped architecture of U-Net. This architecture includes encoder layers, a bottleneck layer, and decoder layers, with subsequent resizing of the patches. A loss function, specifically Sparse Categorical Cross-Entropy, is then defined to evaluate the disparity between predicted masks and labelled data masks. The training process utilised the Adam optimiser to minimise the specified loss function, with hyperparameters tailored for model training. Table 5 provides an overview of the essential parameters and configurations utilised in the development of the model. It summarises the preprocessing procedures, model architectures, and training settings. The preprocessing phase encompasses the patch sizes and overlap settings, as well as the application of low-pass and Gaussian blur filters. The dataset was partitioned into training and testing sets, with specified random state values to ensure reproducibility. Multiple model architectures were employed, each characterised by distinct elements such as the number of convolution layers, kernel sizes, and dropout rates. Additionally, diverse learning rates, batch sizes, and epochs were applied during model training and tuning. The optimal hyperparameters for the U-Net model that yielded the best results included a patch size of 128 × 128 with a 30% overlap, no filters applied, and a train-test split of 25%. Additionally, the optimal configuration involved convolution layers spanning from 64 to 1024, a dropout rate of 0.2, a learning rate set to 0.001, and a batch size of 25. To achieve optimal performance, the model underwent training for 400 epochs.

### 3.3. Verification

Once both algorithms had been trained, the model was applied to the test data set. A series of evaluation metrics were established to ensure that the model was handling the test data well. Evaluation descriptors, including true positive (TP), false positive (FP), true negative (TN), and false negative (FN), were used to estimate the precision, recall, F1-score, and Intersection over Union (IoU). Equation (1) shows the formula for precision, which is the ratio between the correctly labelled pixels and the total count of pixels that were correctly labelled as well as those that were mislabelled within a specific class. Recall, as seen in Equation (2), is the proportion between the correctly labelled pixels and the summation of correctly labelled pixels and pixels that should have been labelled for a particular class but were not. F1-score, Equation (3), is a metric that measures a model’s accuracy. It combines both precision and recall metrics and computes how many times the model correctly predicted a pixel in a class. IoU stands for intersection over union and is a percentage indicating the amount of overlap between the expected number of labelled pixels and the actual area of labelled pixels (Equation (4)). This metric was only applied to the U-Net model. Another validation method for the model is called K-fold cross validation. This is where k number of folds are put into a dataset (split up). For each fold, training is conducted on the remaining folds and the data in the specified fold is used as validation data. This tests the model’s ability to handle new unseen data.

(1) $$\mathrm{Precision}=\frac{\mathrm{TP}}{\mathrm{TP}+\mathrm{FP}}$$

(2) $$\mathrm{Recall}=\frac{\mathrm{TP}}{\mathrm{TP}+\mathrm{FN}}$$

(3) $$F1-\mathrm{score}=\frac{2\mathrm{TP}}{\mathrm{FP}+2\mathrm{TP}+\mathrm{FN}}$$

(4) $$\mathrm{Intersection}\;\mathrm{over}\;\mathrm{Union}\;(\mathrm{IoU})=\frac{\mathrm{Area}\;\mathrm{of}\;\mathrm{intersection}}{\mathrm{Area}\;\mathrm{of}\;\mathrm{Union}}$$

### 3.4. Prediction

After training the XGBoost model, the outcomes were assessed through the application of inference using the trained model. The entire multispectral orthomosaic was cropped into tiles, each measuring 700 × 700 pixels. A batch processing script facilitated the application of inference to individual tiles, which were subsequently assembled in QGIS to generate the final segmented map. For the U-Net model prediction, a different tile size of 128 × 128 pixels was employed. Similarly, to the XGBoost prediction, inference was applied using a batch processing script. The resulting predicted tiles were then stitched together using QGIS, producing the final segmented map for the U-Net model.

## 4. Results

### 4.1. Correlation Analysis and Feature Ranking

**Figure 12:**
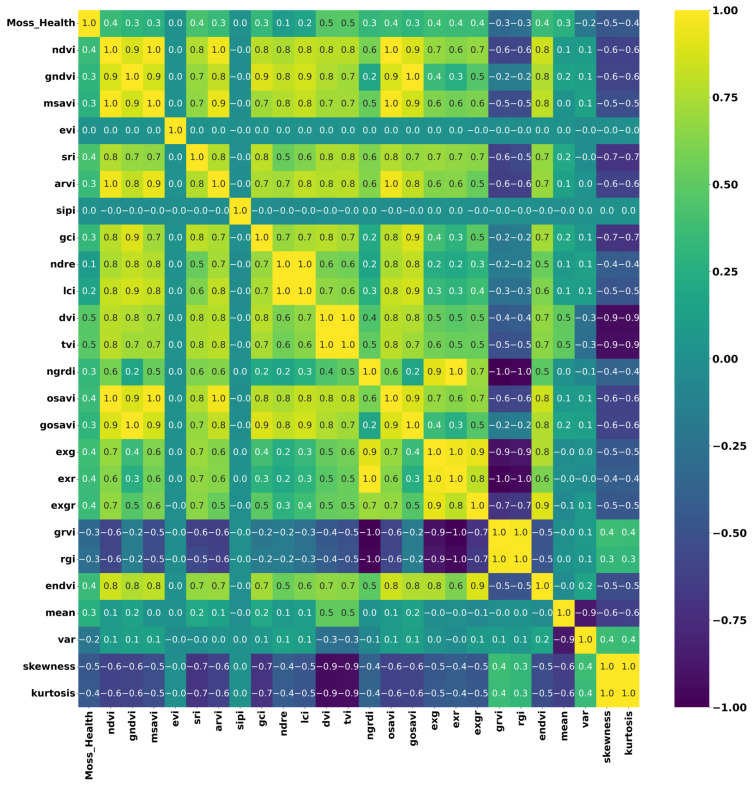
Correlation matrix heatmap depicting the interrelationships among various vegetation indices used in the XGBoost model training for segmenting the healthy condition of moss and lichen. The colour intensity in the heatmap indicates the strength and direction of the correlations between different indices and statistical measures.

Figure 12 illustrates the resultant correlation matrix between moss health and spectral indices. In the results, as depicted in the correlation matrix, no significant correlations, either positive or negative, were observed, with coefficients falling below 0.5, indicating a lack of strong associations between moss health and the analysed spectral features.

**Figure 13:**
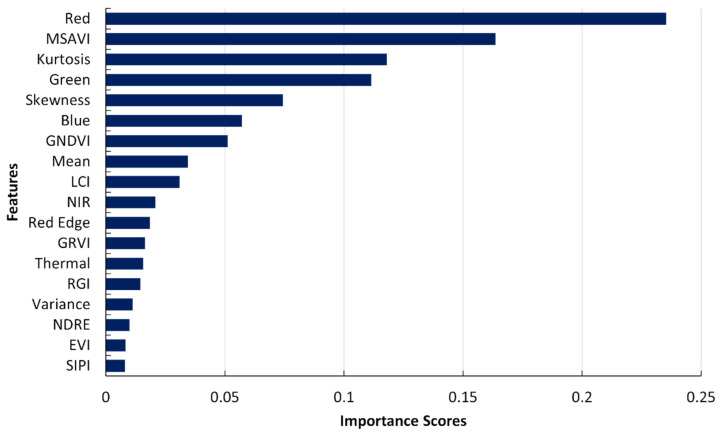
Bar diagram of feature ranking, displaying the importance of different features in the XGBoost model’s prediction.

An additional approach to assess the significance of input features using XGBoost models involves feature ranking, providing the importance of distinct features in influencing the model’s predictions. Figure 13 shows the relative importance of each feature in the predictions for XGBoost. The red band (668), MSAVI, and kurtosis emerged as the top three influential features in the model training process. The second highest spectral index was GNDVI, while SIPI and EVI exhibited comparatively lower importance. It was found that a series of eight spectral indices produced a better model. These eight indices included GNDVI, MSAVI, LCI, GRVI, RGI, NDRE, EVI, and SIPI.

### 4.2. Performance of XGBoost

**Figure 14:**
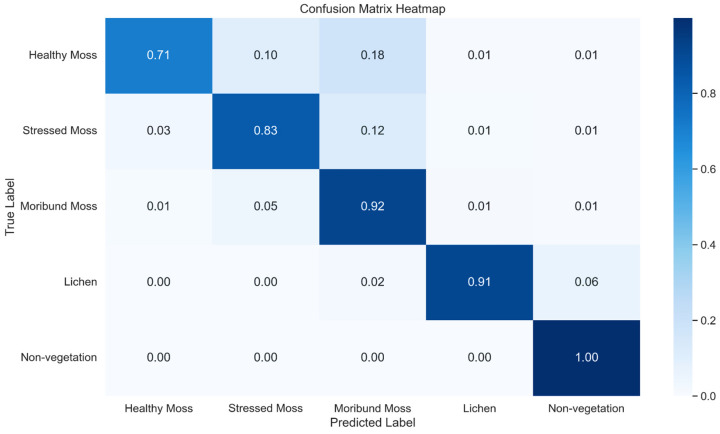
Normalised confusion Matrix Heatmap representing the classification performance of XGBoost across five classes.

The test data was used to verify the model’s capabilities after model training. The classification report (Table 6) demonstrates the model’s ability to predict each class on the test data and Figure 14 demonstrates the normalised confusion matrix for all classes. The precision, recall, and F1-score of the whole model overall were significantly high (91%, 88%, 89% respectively), as this is combining all five classes. When investigating the individual classes, healthy moss had the lowest recall and F1-score. This is because this class had the lowest amount of labelling as seen in the support column. Due to the low number of samples, not just for healthy moss but overall, the model could not perform better than these results without serious overfitting. Though most of the healthy moss pixels are categorised as the correct class, there are a lot of healthy moss pixels labelled as stressed or moribund moss. This is why the recall value was so low and demonstrates that the separability between these classes is very narrow. With the trained model, K-fold cross validation was done using 10 folds, using the scikit-learn Python library. This technique evaluates the accuracy of 10 sample combinations to observe whether the average accuracy of labelled data was similar to the unseen data. The cross-validation results indicate that the average accuracy was 98.5% and the standard deviation was 0.07%.

### 4.3. Performance of U-Net

**Figure 15:**
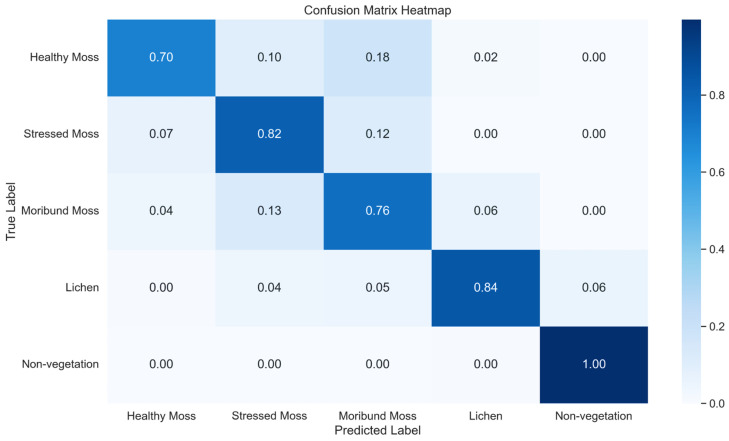
Normalised confusion Matrix Heatmap representing the classification performance of U-Net across five classes using method 1.

We explored two methods for U-Net model development. The first method involves utilising the same labelled data employed in the development of the XGBoost model, while the second method incorporates XGBoost predictions as inputs for the U-Net model, leveraging labelled data for enhanced performance. In Table 7, a comprehensive Classification Report outlines key metrics such as precision, recall, and F1-score for a U-Net model, while Figure 15 visually presents the normalised confusion matrix heatmap, providing a detailed insight into the classification performance across the five classes using method 1.

The second method for U-Net model development yields significant improvements in various performance metrics compared to the first method. Notably, Method 2 demonstrates a marked increase in precision for Healthy Moss by 0.20 (from 0.74 to 0.94), Stressed Moss by 0.17 (from 0.69 to 0.86), and Moribund Moss by 0.10 (from 0.77 to 0.87). In terms of recall, Method 2 shows a slight improvement for Healthy Moss (up 0.01 from 0.70 to 0.71) and substantially better precision for Moribund Moss (up 0.18 from 0.76 to 0.94). The F1-score sees noteworthy enhancements in Healthy Moss by 0.09 (from 0.72 to 0.81), Stressed Moss by 0.11 (from 0.75 to 0.86), and Moribund Moss by 0.13 (from 0.77 to 0.90). Additionally, Intersection over Union (IoU) values exhibit consistent improvements across all classes, with gains in Healthy Moss by 0.11 (from 0.56 to 0.67), Stressed Moss by 0.15 (from 0.60 to 0.75), and Moribund Moss by 0.19 (from 0.63 to 0.82). These quantitative improvements underscore the effectiveness of the second method, leveraging XGBoost predictions for U-Net input, in enhancing the overall performance and accuracy of the model compared to the first method.

**Figure 16:**
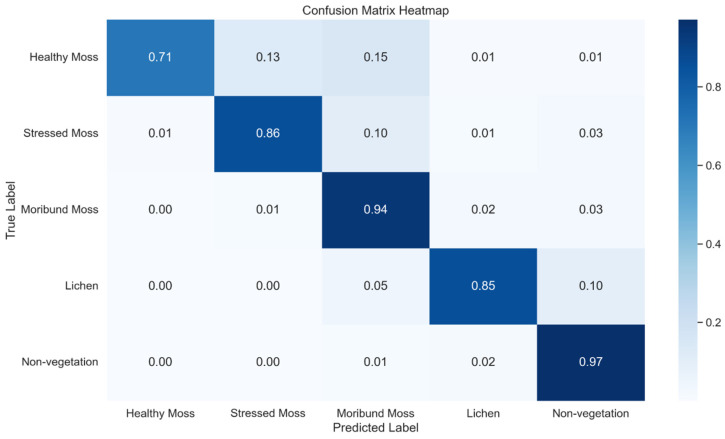
Normalised confusion Matrix Heatmap representing the classification performance of a U-Net across five classes using method 2.

Table 8 outlines the classification report, offering a detailed assessment of the U-Net model’s performance metrics, including precision, recall, and F1-score, across five distinct classes. Notably, the two-stage ensemble approach, incorporating predictions from the initial XGBoost model as inputs for the subsequent U-Net model, yielded a discernible enhancement in overall performance. Figure 16 further elucidates these advancements by presenting a normalised confusion matrix heatmap, visually encapsulating the improved classification performance of the U-Net across the diverse classes. The heatmap serves as a compelling representation of the model’s ability to leverage the complementary strengths of XGBoost, resulting in a refined and more accurate predictive framework. This observed performance boost substantiates the efficacy of the proposed two-stage ensemble methodology in achieving superior outcomes compared to standalone models, underscoring the synergy between XGBoost and U-Net in the context of the specific research objectives.

**Figure 17:**
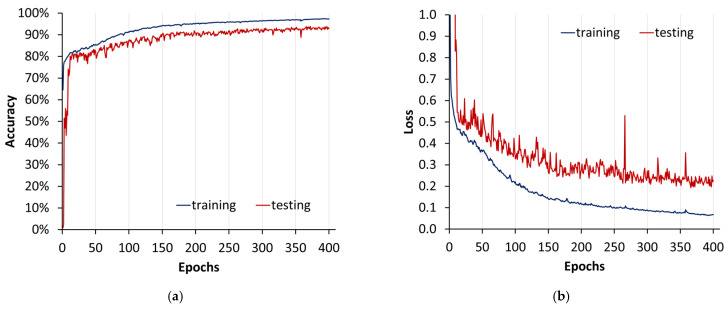
U-Net model training plot from method 2: (**a**) accuracy; (**b**) loss. This visual representation spans epochs 0 to 400, providing an insight into the evolution of the model’s performance over a training period.

The training and validation trends over the first 400 epochs show a positive trajectory in terms of accuracy and a decreasing trend in loss. The model starts with an accuracy of around 64.5% and steadily improves, reaching approximately 96.4% at epoch 400. Concurrently, the training loss decreases from over 6000 to around 0.09, indicating that the model is learning and generalising well. The validation accuracy and loss also exhibit a positive trend, although with some fluctuations. The validation accuracy starts from a very low value (0.008) and increases to approximately 92.8%, while the validation loss decreases from 6192 to around 0.23 (Figure 17). This suggests that the model is performing well on both training and validation datasets, demonstrating good learning and generalisation capabilities. Regular fluctuations in validation metrics might indicate a degree of overfitting, and further analysis, such as model fine tuning or the use of regularisation techniques, could be explored to enhance generalisation.

**Figure 18:**
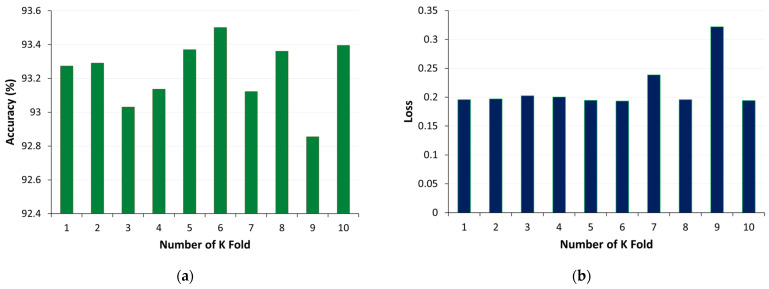
K-fold cross validation for U-Net model using method 2: (**a**) accuracy, (**b**) loss.

Figure 18 provides an overview of the K-fold cross-validation results for the U-Net model. Figure 18a displays the accuracy of the model across different folds, demonstrating its consistency and performance, while Figure 18b visualises the loss, indicating the model’s error rate during cross validation. Additionally, the model’s overall performance across all folds is summarised, with an overall accuracy of approximately 93.23% with a standard deviation of 0.19 and a loss of approximately 0.21 with a standard deviation of 0.04.

### 4.4. Segmented Maps

**Figure 19:**
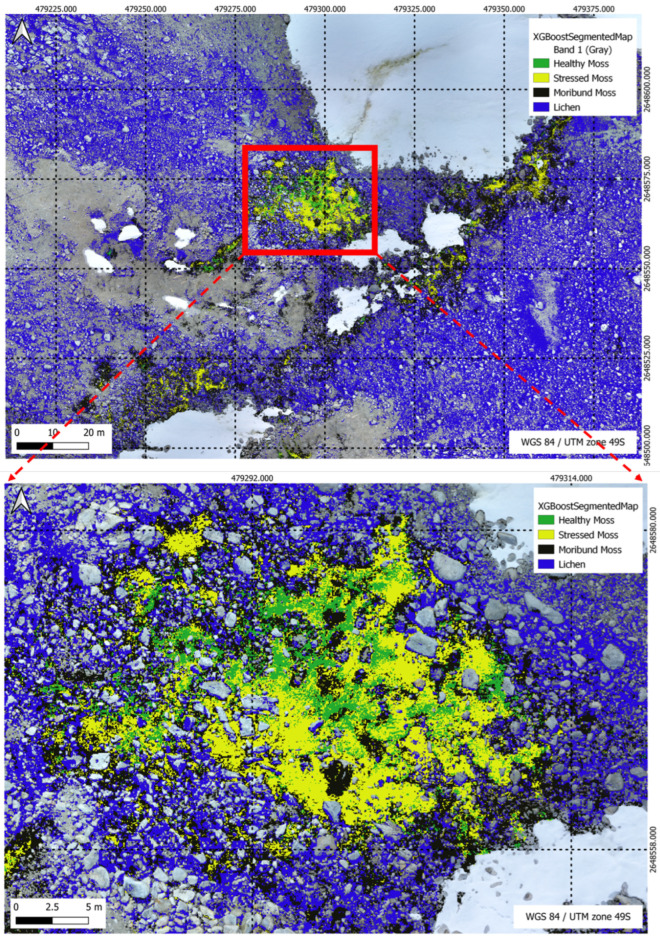
XGBoost Segmentation results for a multi-class scenario, highlighting the model’s classification outcomes across five distinct classes in the segmentation task.

**Figure 20:**
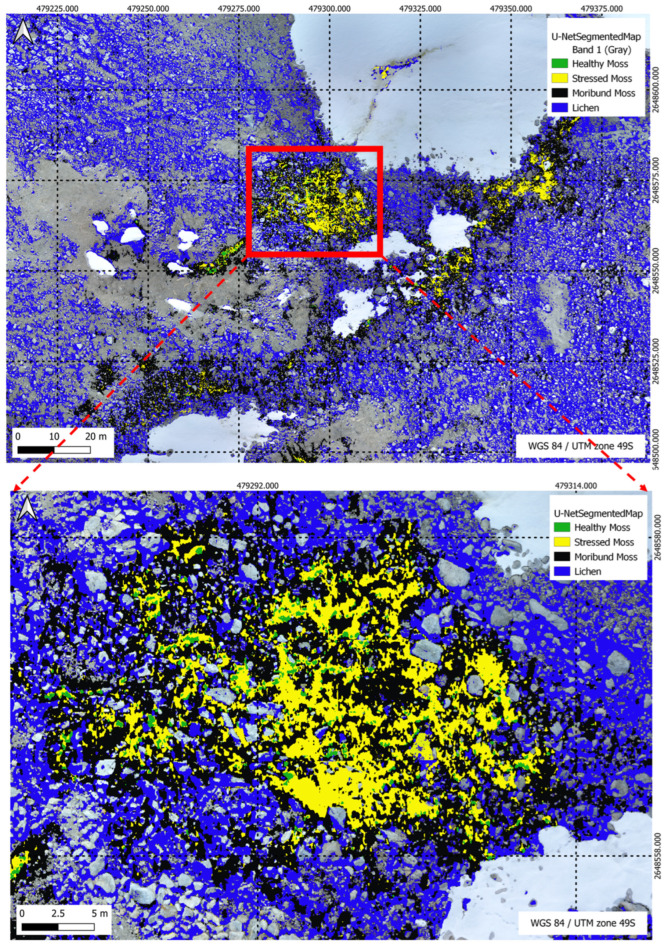
U-Net Segmentation results (method 2) for a multi-class scenario, highlighting the model’s classification outcomes across five distinct classes in the segmentation task.

In Figure 19, the XGBoost Segmentation results unfold, showcasing the model’s classification outcomes with precision across five distinct classes in the complex segmentation task. Meanwhile, Figure 20 delves into the U-Net Segmentation results using method 2, offering a detailed perspective on the model’s classification outcomes across the same five distinct classes in the segmentation task.

**Figure 21:**
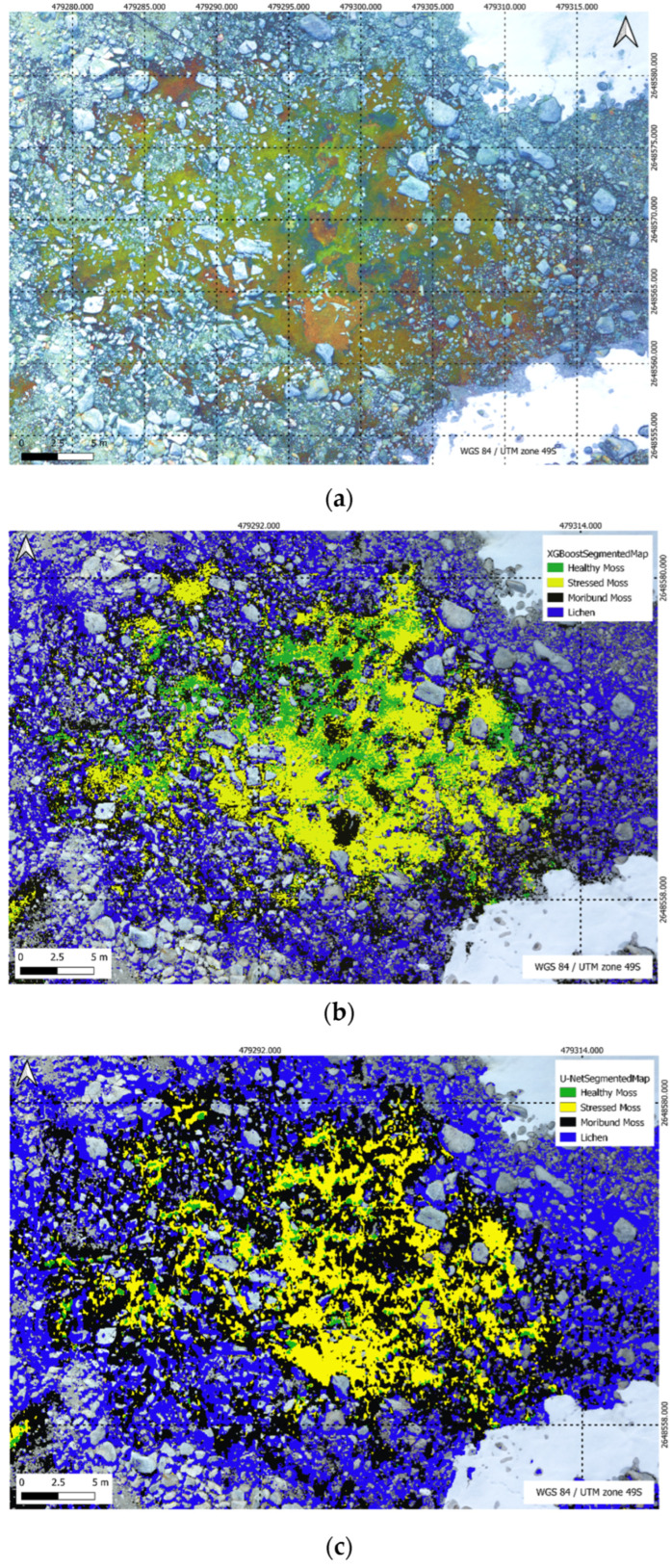
(**a**) High resolution RGB image; (**b**) U-Net segmentation; (**c**) XGBoost segmentation.

Figure 21 showcases key visual outputs, including (a) a high-resolution RGB image, (b) U-Net segmentation, and (c) XGBoost segmentation.

Table 9 illustrates the percentage distribution of vegetation classes within the specified region of 931.12 m^2^ as shown in Figure 19, comparing the performance of the XGBoost and U-Net models. Healthy moss, stressed moss, moribund moss, and lichen are the identified classes, highlighting the varying proportions of each class as predicted by the respective models.

**Figure 22:**
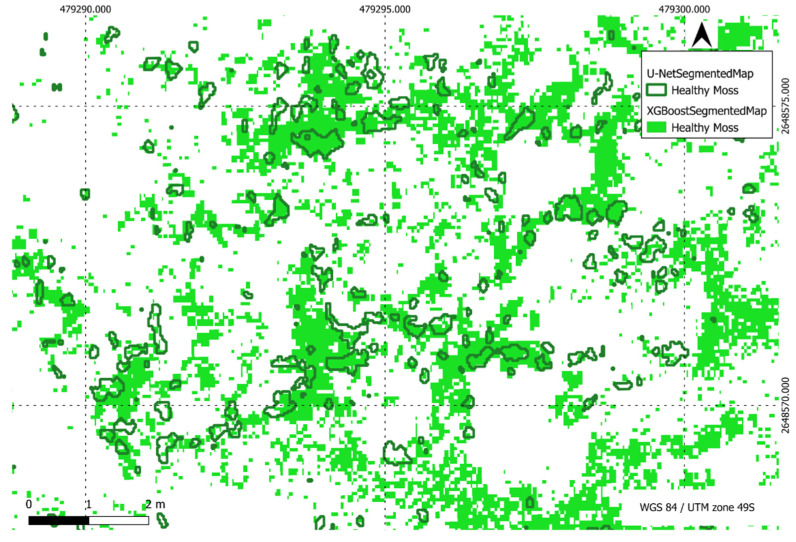
Comparison of XGBoost and U-Net predictions of healthy moss in a region of interest.

**Figure 23:**
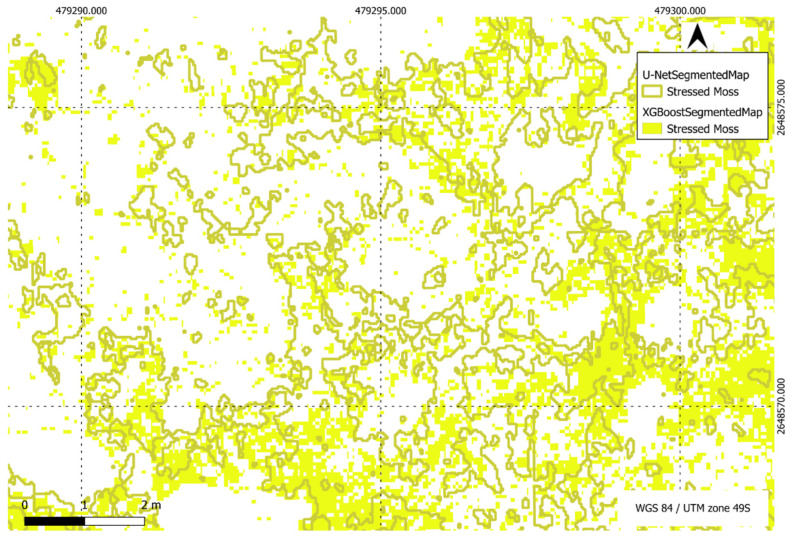
Comparison of XGBoost and U-Net predictions of stressed moss in a region of interest.

**Figure 24:**
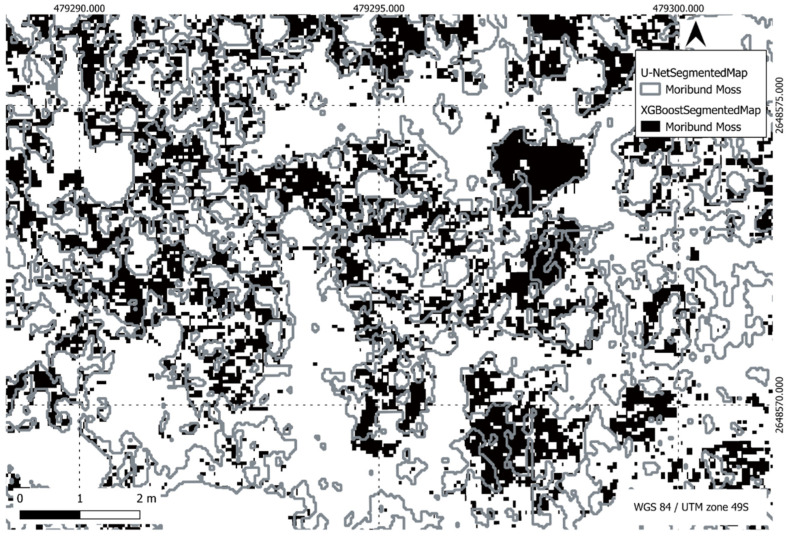
Comparison of XGBoost and U-Net predictions of moribund moss in a region of interest.

**Figure 25:**
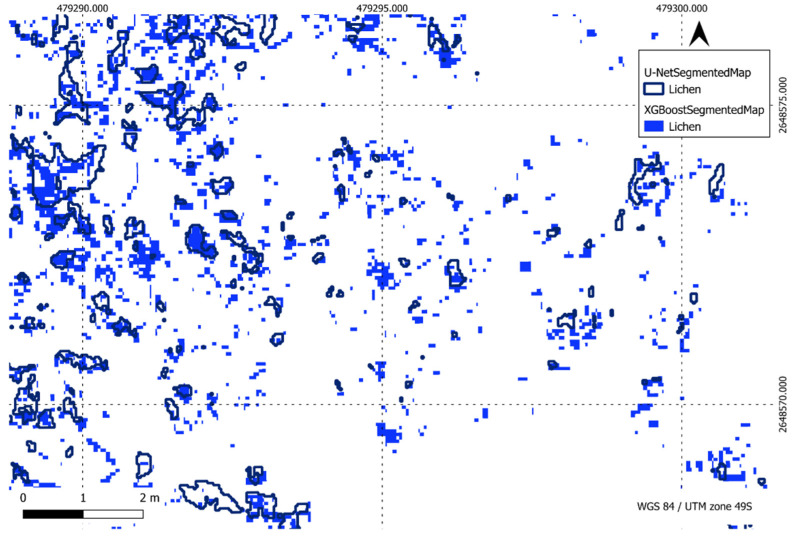
Comparison of XGBoost and U-Net predictions of lichen in a region of interest.

Figure 22, Figure 23, Figure 24 and Figure 25 present a visual comparison between XGBoost predictions and U-Net predictions within a defined ROI. The visualisation allows for a detailed examination of the intersection and union between the two models. The overlay of XGBoost predictions over U-Net predictions provides valuable insights into the complementary strengths and weaknesses of the two approaches, contributing to a comprehensive understanding of their performance in the specific targeted region.

## 5. Discussion

The purpose of this paper was to verify a methodology of using multispectral imagery, UAVs, and ML to classify moss health (healthy, stressed, and moribund) and lichen in Antarctica. The methodology and segmented maps of ASPA 135 will help scientists to perform non-invasive field tests to monitor the health of moss beds. The physical risk to the moss during ground testing plus the labour demand is extremely high; therefore, the utilisation of UAVs to conduct continuous monitoring will promote sustainability and reduce future costs. The XGBoost model performed well with the limited range of samples. With an F1-score of 89%, the model was able to accurately segment moss health and lichen out of the orthomosaic. Eischeid et al. had an F1-score of 85% for the Random Forest (RF) algorithms used for disturbance mapping on tundra vegetation in the Artic [64]. Likewise, Sotille et al. used an RF classifier on Antarctic vegetation and got an accuracy of 96.6% [65]. This supports the findings of Sotille et al. and Turner et al. as more ground truth data would have allowed the RF model to be more transferable to new data [66]. In addition, more ground truth data for the XGBoost model would have helped the labelling process as the manual polygons would have been less subjective.

In comparison to prior studies employing semi-automatic object-based image analysis (OBIA) for moss health classification in the same moss beds over the 2003–2013 timeframe, our research introduces a pioneering approach [4]. While the conventional OBIA method achieved 84% accuracy within fixed quadrat locations (25 cm × 25 cm), our methodology utilises drone-based multispectral imaging and DL, enabling a larger spatial coverage and automated classification. The key distinction lies in our ability to cover expansive areas, providing a more comprehensive understanding of moss health dynamics. Additionally, the integration of multispectral imagery enhances spectral resolution, contributing to a refined and detailed classification of moss health. This novel approach represents a significant advancement in the field, offering a more efficient and accurate means of assessing moss health and mapping over larger territories.

In this study, a two-stage ensemble methodology was employed for predictive modelling, leveraging the strengths of XGBoost and a U-Net architecture. In the initial stage, XGBoost, a robust gradient-boosting algorithm, was utilised for making predictions on the target variable. The outputs generated by XGBoost were subsequently employed as input features for a U-Net model in the second stage. The U-Net, renowned for its effectiveness in image segmentation tasks, harnessed the information encoded by XGBoost to refine and enhance the predictive capabilities. This two-step modelling approach, involving the sequential utilisation of XGBoost and U-Net, aimed to capitalise on the complementary strengths of the two algorithms, potentially yielding improved predictive performance compared to standalone models. The methodology encapsulates a novel strategy for ensemble modelling, strategically incorporating the distinctive attributes of each model to achieve a more robust and accurate predictive framework.

In the context of our research, the integration of the U-Net model with XGBoost predictions is a novel approach designed to enhance segmentation accuracy. We acknowledge the potential concern regarding the accuracy of initial XGBoost predictions and its influence on subsequent U-Net segmentation. To address this, our methodology incorporates data preprocessing (selection of tiles), parameter tuning (dropout, learning rate, and different convolution layer), and model training strategies (batch size and epochs) to minimise the impact of incorrect predictions. We emphasise the importance of transparently discussing these challenges and limitations. Additionally, our experimental validation includes a thorough assessment of model performance under various conditions (k-fold cross validation), demonstrating the robustness and generalisability of our integrated methodology. This proactive approach to addressing potential errors ensures a comprehensive understanding of the model’s capabilities and limitations in real world applications.

The finer details of the moss could not be identified because of the resolution of the Altum multispectral camera. The fidelity displayed in the Mini 3 Pro could not be replicated in the XGBoost model because of the lower resolution. Higher resolution data would allow for a more intricate segmented map, which would be more accurate as the moss classes are not constrained to particular patches but are intertwined across the ASPA 135 landscape. Also, higher resolution would have helped the labelling process. Turner et al. concluded that imagery of 3cm/pixel was suitable enough for vegetation detection classification [66]. This paper found that having a higher resolution was necessary, especially when labelling non-ground truth data points. The Mini 3 Pro dataset is a very small orthomosaic; having access to the same high resolution all over ASPA 135 would have allowed labelling across a much larger area, meaning better results. This would also have helped the U-Net architecture. Sotille et al. came to the same conclusion when using GEOBIA to classify maritime Antarctic vegetation. However, labelling such an extensive data set involves a lot of manual work [64].

Our study focuses on evaluating the health of moss and lichen using multispectral imagery captured by UAVs, employing ML classifiers such as XGBoost for segmentation. In contrast, another study by Sandino et al. primarily addresses the challenge of mapping the same study location using a workflow that integrates UAV, hyperspectral imagery, and same ML classifiers of XGBoost [39]. This approach resulted in an average accuracy of 95%, demonstrating the successful detection and mapping of moss and lichens. While both studies leverage ML, the first emphasises health assessment, and the second focuses on precise mapping, yet both contribute valuable insights into the potential applications of remote sensing technologies in monitoring the impact of climate change on the Antarctic ecosystem. Despite our adoption of the U-Net model in this study, the achieved results were comparatively lower than those obtained in the hyperspectral study that utilised XGBoost [39]. One prominent factor contributing to this performance gap is the insufficient number of training samples available for U-Net. In ML, particularly for DL models like U-Net, the quantity and diversity of training data play a crucial role in model performance. The U-Net model requires a substantial volume of diverse training samples to effectively learn and generalise patterns within the data. In our case, the limited availability of training samples likely hindered the U-Net model’s ability to discern complex spectral patterns and variations associated with the health assessment of moss and lichen. Shortage of diverse training examples can result in suboptimal model performance, as the model struggles to capture the full range of features necessary for accurate segmentation. This shortage of diverse training examples can result in suboptimal model performance, as the model may struggle to capture the full range of features necessary for accurate segmentation. On the other hand, the XGBoost model employed in the hyperspectral study benefited from a more extensive dataset, allowing it to better learn the intricate relationships between spectral characteristics and vegetation classes. The abundance of training samples facilitated the XGBoost model’s ability to generalise and make accurate predictions, ultimately contributing to the higher overall performance observed in the hyperspectral study.

The utilisation of UAVs in monitoring moss and lichen communities in East Antarctica presents numerous advantages, underscoring its potential as a valuable tool for ecological research in challenging environments. The high spatial resolution and multispectral capabilities of UAVs allow for detailed and accurate vegetation assessments, offering insights into the health and dynamics of moss and lichen communities. The non-invasive nature of UAV-based data collection minimises disturbance to the delicate Antarctic ecosystems, providing an environmentally sensitive approach to monitoring. However, it is imperative to address certain limitations inherent in UAV-based studies. One notable constraint is the limited endurance of UAV flights, restricting the coverage area per mission. This limitation may necessitate multiple flights to adequately survey larger expanses, potentially leading to increased logistical complexities and resource requirements. Moreover, the payload capacity of UAVs may impose restrictions on the types of sensors and equipment that can be deployed, influencing the comprehensiveness of data collection.

Another critical consideration is the susceptibility of UAV operations to adverse weather conditions prevalent in Antarctica. Unpredictable weather patterns, including strong winds, extreme cold, and snow coverage, can impede flight schedules and data collection efforts. Ensuring reliable and consistent data acquisition becomes particularly challenging in such extreme environmental conditions. Despite these challenges, the study underscores the transformative potential of UAV technology in advancing non-invasive monitoring techniques for polar ecosystems. The combination of high-resolution imagery and ML classifiers facilitates an understanding of vegetation health, contributing to biodiversity conservation efforts in remote and inaccessible regions. Future advances in UAV technology, including increased flight endurance and enhanced weather resilience, hold promise for overcoming current limitations and further expanding the applicability of UAVs in polar ecological research.

## 6. Conclusions and Recommendations

This paper presented a novel workflow that combined UAVs, multispectral and RGB imagery, and ML to monitor the health of Antarctic vegetation, validated with a case study for detection and mapping of moss and lichen from a dataset collected at ASPA 135 in early 2023. This study addressed a research gap found in previous works by comparing two state-of-the-art ML classifiers: XGBoost and U-Net. The XGBoost model demonstrated robust classification across various vegetation classes, showcasing high precision, recall, and F1-score. The U-Net model, while adopting different segmentation methods, presented different outcomes. Method 1 showed moderate performance, indicating potential limitation of lack of training pixels to classify the health status of moss. In contrast, Method 2 illustrates improvements; the U-Net model’s performance is influenced by the availability of training samples, underscoring the importance of the segmentation approach. The choice between XGBoost and U-Net should consider the quantity of training samples because DL models like U-Net require more training samples than XGBoost. However, the collection of more ground truth information to increase the training samples from the Antarctic is a challenging task. Therefore, one of the techniques to overcome this challenge is to use classical ML like XGBoost for initial predictions which can then be used as inputs to DL models. This presents a viable strategy to address this challenge.

Further exploration and refinement of ML techniques tailored to polar environments will advance our understanding and monitoring capabilities in these critical ecosystems. Future areas of work include capturing more high-resolution RGB data for increased labelling to enhance the CNN classifier performance and to visually verify polygon labelling. Additionally, higher resolution multispectral data should be captured to create more accurate segmented maps that mimic the dispersed characteristics of vegetation classes in nature. Detailed analysis on which spectral indices impact what class should be investigated. Furthermore, evaluation on other ML algorithms could improve the segmentation of Antarctic vegetation. The use of UAVs in Antarctica is still relatively new; however, it has effectively become the most non-invasive method to monitor the health of these fragile ecosystems.

## Figures and Tables

**Table 1:** Key specifications of different aircraft used in this study.

Specifications	BMR3.9RTK	DJI Mini 3 Pro
Weight	12 kg, Maximum take-off weight of 14 kg	Less than 249 g
Battery type	Two six cell LiPo batteries	Li-ion
Flight time	30 min with dual payload capability	34 min (with Intelligent Flight Battery measured while flying at 21.6 kph in windless conditions)

**Table 2:** Key specifications of different sensors used in this study.

Specifications	MicaSense Altum	Sony Alpha 5100	DJI Mini 3 Pro Inbuilt Camera
Number of bands	Five multispectral and a thermal band	Three	Three
Bands	blue, green, red, red-edge, and near-infrared, short wave infrared (thermal)	blue, green, red	blue, green, red
Resolution	Multispectral: 3.2 megapixels (MPs) and Thermal: 320 × 256 pixels.	24.3 MPs	48 MPs
Field of view	50.2° × 38.4°	83°	82.1°

**Table 3:** Comprehensive overview of moss health states and descriptions.

Health Status of Moss	Description
Healthy	Moss in good health exhibits shades ranging from dark (ms_gr) to bright green (ms_bg), indicating healthy chloroplasts and plenty of chlorophyll. It is typically found in regions with consistent meltwater availability.
Stressed	Moss undergoing stress experiences a reduction in chlorophyll pigments, appearing red or brown (ms_rd) due to the presence of carotenoids and other photoprotective pigments, as noted by Waterman et al. (2018). Stressors such as drought or intense solar radiation can contribute to this condition. However, if the stress is reversed, the moss turf has the potential to regain its green colour, facilitated by the formation of new leaves [4].
Moribund	Intense or prolonged stress leads to the moribund state of moss, where leaves undergo pigment loss, rendering them grey in colour (ms_bw and ms_mm). Additionally, these stressed moss specimens may become encrusted with lichens.

**Table 4:** Comprehensive overview of the various spectral indices utilised in the current study, offering a detailed compilation of the specific indices employed to analyse and interpret spectral data.

Spectral Indices	Formula	Literature Review
Normalised Difference Vegetation Index (NDVI)	$\frac{NIR-R}{NIR+R}$	[43]
Green Normalised Difference Vegetation Index (GNDVI)	$\frac{NIR-G}{NIR+G}$	[44,45,46]
Modified Soil-Adjusted Vegetation Index (MSAVI)	$\frac{2\times NIR+1-\sqrt{2\left(2\times NIR+1\right)-8(NIR-R)}}{2}$	[47]
Enhanced Vegetation Index (EVI)	$\frac{2.5(NIR-R)}{NIR+6R-7.5B+1}$	[48]
Simple Ratio Index (SRI)	$\frac{NIR}{R}$	[49]
Atmospherically Resistant Vegetation Index (ARVI)	$\frac{NIR-(R-2\left(B-R\right))}{NIR+(R-2\left(B-R\right))}$	[47]
Structure Insensitive Pigment Index (SIPI)	$\frac{NIR-B}{NIR-R}$	[50]
Green Chlorophyll Index (GCI)	$\frac{NIR}{G}-1$	[51,52]
Normalised Difference Red Edge Index (NDRE)	$\frac{NIR-RedEdge}{NIR+RedEdge}$	[53,54,55]
Leaf Chlorophyll Index (LCI)	$\frac{NIR-RedEdge}{NIR+R}$	[48]
Difference Vegetation Index (DVI)	NIR−R	[46,51,56]
Triangular Vegetation Index (TVI)	60(NIR−G)−100(R−G)	[48]
Normalised Green Red Difference (NGRDI)	$\frac{G-R}{G+R}$	[46,57]
Optimised Soil-Adjusted Vegetation Index (OSAVI)	$\frac{1.16(NIR-R)}{NIR+R+0.16}$	[58]
Green Optimised Soil Adjusted Vegetation Index (GOSAVI)	$\frac{NIR-G}{NIR+G+0.16}$	[58]
Excess Green (EXG)	$\frac{2G-R-B}{R+G+B}$	[59]
Excess Red (EXR)	$\frac{1.4R-G}{R+G+B}$	[59]
Excess Green Red (EXGR)	ExG−ExR	[59]
Red Green Index (RGI)	$\frac{R}{G}$	[59]
Green Red Vegetation Index (GRVI)	$\frac{R-G}{R+G}$	[59]
Enhanced Normalised Difference Vegetation Index (ENDVI)	$\frac{(NIR+G)-2B}{\left(NIR+G\right)+2B}$	[50]

R: Red; G: Green; B: Blue: NIR: Near Infra-Red.

**Table 5:** Parameter tuning for U-Net training.

Preprocessing	Patch size	32, 64, 128, 256
	Overlap	0.1, 0.2, 0.3
	Low pass filter	Without filter, 3 × 3, 5 × 5, 7 × 7
	Gaussian blur filter	Without filter, 3 × 3, 5 × 5, 7 × 7
	Train test split	20%, 25%, 30%
Model Architecture	Convolution layers	8–1024, 16–1024, 32–1024, 64–1024, 128–1024, 16–512, 32–512, 128–512, 8–256, 16–256, 32–256
	Kernal size	1 × 1, 3 × 3, 5 × 5
	Dropout	0.1, 0.2
Model compile and Training	Learning rate	0.1, 0.01, 0.001, 0.0001
	Batch size	10, 15, 20, 25, 30, 35
	Epochs	50, 75, 100, 150, 200, 250, 300, 400

**Table 6:** Classification Report summarising key metrics for an XGBoost model, including precision, recall, and F1-score, across five classes.

Classes	Precision	Recall	F1-Score
Healthy Moss	0.86	0.71	0.78
Stressed Moss	0.86	0.83	0.85
Moribund Moss	0.88	0.92	0.90
Lichen	0.94	0.91	0.93
Non-Vegetation	1.00	1.00	1.00

**Table 7:** Classification Report summarising key metrics for a U-Net model, including precision, recall, and F1-score, across five classes using method 1.

Classes	Precision	Recall	F1-Score	IoU
Healthy Moss	0.74	0.70	0.72	0.56
Stressed Moss	0.69	0.82	0.75	0.60
Moribund Moss	0.77	0.76	0.77	0.63
Lichen	0.86	0.84	0.85	0.74
Non-Vegetation	1.00	1.00	1.00	0.99

**Table 8:** Classification Report summarising key metrics for a U-Net model, including precision, recall, and F1-score, across five classes using method 2.

Classes	Precision	Recall	F1-Score	IoU
Healthy Moss	0.94	0.71	0.81	0.67
Stressed Moss	0.86	0.86	0.86	0.75
Moribund Moss	0.87	0.94	0.90	0.82
Lichen	0.95	0.85	0.90	0.82
Non-Vegetation	0.94	0.97	0.96	0.92

**Table 9:** Class distribution in the target area (931.12 m^2^) using XGBoost and U-Net Models.

Class	XGBoost	U-Net
Healthy Moss	6%	3%
Stressed Moss	15%	12%
Moribund Moss	20%	30%
Lichen	21%	21%

## Data Availability

Research data can be made available upon request to the corresponding author.
